# An auditory brain-computer interface to detect changes in sound pressure level for automatic volume control

**DOI:** 10.1016/j.heliyon.2023.e23948

**Published:** 2023-12-18

**Authors:** Riki Kimura, Isao Nambu, Rui Fujitsuka, Yoshiko Maruyama, Shohei Yano, Yasuhiro Wada

**Affiliations:** a Graduate School of Engineering, Nagaoka University of Technology, 1603-1 Kamitomioka, Nagaoka, Niigata, 940-2188, Japan; b Department of Production Systems Engineering, National Institute of Technology, Hakodate College, 14-1 Tokura, Hakodate, Hokkaido, 042-8501, Japan; c Department of Electrical and Electronic Systems Engineering, National Institute of Technology, Nagaoka College, 888 Nishikatakai, Nagaoka, Niigata, 940-8532, Japan

**Keywords:** BCI, Oddball, Automatic volume control, Sound level

## Abstract

Volume control is necessary to adjust sound levels for a comfortable audio or video listening experience. This study aims to develop an automatic volume control system based on a brain-computer interface (BCI). We thus focused on a BCI using an auditory oddball paradigm, and conducted two types of experiments. In the first experiment, the participant was asked to pay attention to a target sound where the sound level was high (70 dB) compared with the other sounds (60 dB). The brain activity measured by electroencephalogram showed large positive activity (P300) for the target sound, and classification of the target and nontarget sounds achieved an accuracy of 0.90. The second experiment adopted a two-target paradigm where a low sound level (50 dB) was introduced as the second target sound. P300 was also observed in the second experiment, and a value of 0.76 was obtained for the binary classification of the target and nontarget sounds. Further, we found that better accuracy was observed in large sound levels compared to small ones. These results suggest the possibility of using BCI for automatic volume control; however, it is necessary to improve its accuracy for application in daily life.

## Highlights

• Electroencephalography was measured during an auditory oddball experiment.
• Event-related potentials were observed when volume was changed as target stimulus.
• Higher classification accuracy for detecting target/nontarget stimuli was observed.
• Automatic volume control using a brain-computer interface may be achieved.

## 1 Introduction

Recently, several video and music streaming services have become available and are commonly used worldwide. While these are easy for listening to or viewing content, the user is required to adjust the audio volume of the equipment when there are differences in volume between the video or music content. To adjust the volume, it is necessary for listeners to move their bodies; therefore, it may be more comfortable if the sound volume is controlled without such adjustment. Additionally, an automatic volume control system may be of interest to individuals who are unable to adjust the volume manually due to injury or disease. Currently, a normalization method based on the equal-loudness contour defined by ISO 226 is commonly used for audio content [1]; however, the equal-loudness contour is calculated based on statistics, and sometimes deviates from the actual sound levels for an individual. Thus, an individualized method for automatic volume control is required.

To this end, we proposed a system that controls the volume of an external device using brain activity measured by an electroencephalogram (EEG), without physical adjustment; this system is called a brain-computer interface (BCI) [2]. The BCI is expected to be an alternative device used to interact with the external world among individuals who have difficulty moving their bodies, such as those with amyotrophic lateral sclerosis. One of the main BCI experimental paradigms is the oddball paradigm [3], wherein external stimuli are continuously presented, and the participant is asked to pay attention to infrequent stimuli. In response to infrequent stimuli, specific event-related potentials (ERPs) are observed. Many different types of stimuli have been examined in the oddball paradigm [4,5]. For example, visual BCIs using flash or color stimuli [3,4], and auditory BCIs using sound direction or frequencies have been proposed [[6], [7], [8]]. However, these stimuli are not used for BCI toward automatic volume control, as they may lose a sense of immersion owing to the attention paid to the stimuli, apart from the main contents. To avoid this, it is considered that the sound volume level itself can be used for the stimuli of the oddball paradigm.

The oddball paradigm was previously examined using sound pressure levels [8]; this previous study conducted a basic study for oddball experiments using sound level. They reported that the classification accuracy and information transfer rate using changes in sound level were degraded when compared with changes in the frequency or direction of sounds; however, two types of sound were used: beeping as the target/nontarget stimuli, and pink noise as the frequent stimuli. This differs from using the sound level of the content as the target/nontarget stimuli; thus, no studies have directly tested the possibility of using a BCI for automatic volume control.

Here, we aimed to examine a BCI for automatic volume control using the same type of stimuli (white noise) with different sound pressure levels for the target, nontarget, and frequent stimuli. We then examined whether different sound levels could be detected using the oddball paradigm. In the first experiment (1-Target), we prepared two types of sound with different levels; one sound (infrequent, 70 dB) was considered the target stimulus. In this simple setting, we examined whether it is possible to detect specific sound levels that the participant could focus on. In the second experiment (2-Target), we prepared three different sound levels and changed the target sound levels in different sessions. Thus, we examined whether it is possible to detect two types of sound levels (i.e., large or small) depending on the experimental setting for application in daily life. We further analyzed the effect of reducing the number of channels and a half-session analysis on real applications in the future. While these investigations are not sufficient to establish automatic volume control using brain signals yet, it may be important step to achieve a future application. The remainder of this paper is organized as following. The materials and methods describe the experimental procedures and data analyses. The results section describes the ERPs and classification accuracy for the two experiments. Finally, the interpretation of the results, limitations, and future work are discussed in the discussion section.

## 2 Materials and methods

### 2.1 Participants

Ten healthy males with no history of hearing impairment participated in this study. After being informed regarding the experiment, they agreed to participate and provided informed consent. Seven of the 10 participants joined both the first and second (1-Target and 2-Target) experiments; the remaining patients only joined the second experiment (2-Target). We included additional participants exclusively for the second experiment, as we initially anticipated potential task difficulty and accuracy variability. The experiment was performed in accordance with the Declaration of Helsinki and approved by the ethical committee of Nagaoka University of Technology (No. R3-12).

### 2.2 Experimental setting

**Fig. 1:**
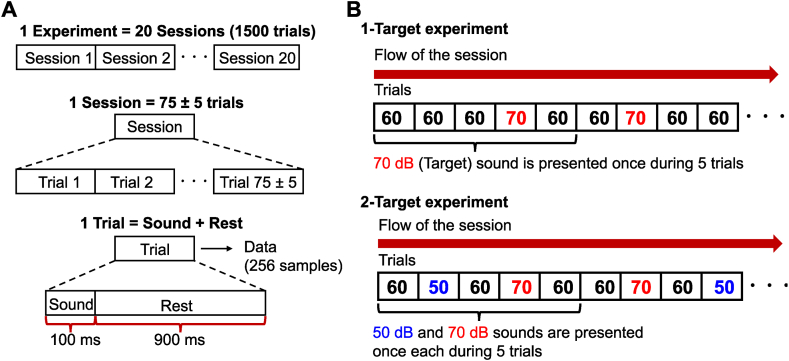
Experiment (A) The experiment comprised 20 sessions, with each session comprising several trials; a single trial included a sound and rest period. (B) Task procedure. In the 1-Target experiment (upper panel), 70 dB sound was defined as the target. The number in the box shows sound volume levels (60 or 70 dB). In the 2-Target experiment (lower panel), 50 dB sound was included and considered a target in the latter half of the sessions.

The participant sat on a chair in a soundproof room, which was completely separated from the operator. The sound stimuli were presented using stereo earphones (ER4SR; Etymotic Research, Illinois, United States). White noise stimuli were generated by a computer and played using a Digital-to-Analog converter (UA-55; Roland Corporation, Shizuoka, Japan) and an analog headphone amplifier (AT-HA21; Audio-Technica Corporation, Tokyo, Japan). The sampling frequency and resolution (bit rate) were 44.1 kHz and 16 bits, respectively. The sound pressure level was defined as the value of a calibrated sound-level meter (Type 6030; ACO Co., Ltd., Tokyo, Japan); for calibration, a dummy-head (Samurai hats, ACO Co., Ltd., Japan) and 1/2 microphone (Type 7013; ACO Co., Ltd., Japan) were also used. The microphone was connected to the sound-level meter and positioned at the eardrum of the dummy head. Sound was then generated from an earphone that was set to the ear of the dummy head, and the sound pressure level was measured. Each experiment comprised 20 sessions, including 70–80 trials each (Fig. 1A). Each trial was 1-s, comprising a 100-ms auditory stimulus and 900-ms rest period (no sound). To use the same amount of data, we discarded data after 70 trials; participants were not informed of this beforehand to avoid influences of concentration during the experiment. Additionally, participants rested for a few minutes every five or 10 sessions.

### 2.3 Task of the two experiments

Two experiments were conducted; in the first (1-Target), we measured the EEG data while white noise stimuli of 60 dB or 70 dB were presented to the participant (Fig. 1B) pseudorandomly. The participant was asked to close their eyes and count the number of presentations of the target stimulus. The target stimulus was set to 70 dB white noise (i.e., a large sound level), which was conveyed to the participant before the session. The sound volume level of 80 % of the presentation stimuli was 60 dB, while the rest of the stimuli (20 %) was 70 dB. We selected 70 dB as the maximum sound pressure level taking into consideration the safety of the participants (the participants were usually uncomfortable with levels above 70 dB). Subsequently, 60 dB was set as a standard sound level, which is 10 dB lower than the maximum sound level. To ensure discernible differences, we maintained a consistent 10 dB gap between the levels, leading us to choose 50 dB as the lower sound level for the second experiment.

In the second (2-Target) experiment, the protocol was almost the same as in the 1-Target experiment; however, low volume level sounds (50 dB) were incorporated. White noise stimuli of 50 dB, 60 dB, or 70 dB were presented to the participant (Fig. 1B) pseudorandomly. Each sound level had a 10 dB gap between each other so that the participant could discern the level difference. In the first half of sessions 1–10, the target stimulus was set to 70 dB white noise, the same as in the 1-Target experiment. In the latter half of sessions 11–20, the target stimulus was 50 dB white noise (i.e., a small sound level). Throughout the sessions, 60 dB stimuli were defined as frequent stimuli, and the participant was asked to ignore it. The percentage of the stimulus type was 20 % for 50 dB, 20 % for 70 dB, and 60 % for 60 dB.

### 2.4 EEG measurement

EEG data were measured using an active gel EEG system (ActiveTwo; Biosemi, Amsterdam, Netherlands). The 64 electrodes were positioned according to the extended international 10–20 systems, and the voltage difference between each electrode and the common mode sense electrode attached to the right earlobe were digitally measured at a sampling rate of 256 Hz (please refer to Biosemi web reference for detail [9]). The measured EEG data and auditory stimuli were synchronized by a trigger input using a parallel port, and the data were clipped and saved for each trial. While the timing of auditory stimuli included a subtle delay due to the buffering of audio devices or programming for generating sounds, it was not influenced by further analysis.

### 2.5 Preprocessing

The same preprocessing technique was applied to the EEG data for both experiments. First, the first five trials after starting each session were removed to avoid the effects of the startle reaction; Second-order Butterworth band-pass filtering of 0.1–8 Hz was then applied to the data. Subsequently, data for each trial (1-s, 256 samples; Fig. 1A) were extracted from time-series data in each session. Next, data from −100 to 0 ms before the start of sound presentation were defined as baseline, and the average of the baseline was subtracted from all the data for baseline correction in each trial. Data after baseline correction were used for ERP analysis. For classification analysis, data after baseline correction were down-sampled to 32 Hz, and data from all channels were concatenated into a vector as an input of classification. The preprocessing was performed by Matlab (version 7.4, Mathworks, Natick, USA).

### 2.6 ERP analysis

To visualize and understand the evoked responses to the stimulus, we performed ERP analysis. To demonstrate the difference between trial types (target and nontarget), we first averaged the time-series EEG data across participants and trials for each target and nontarget condition, and examined the temporal profile of ERPs in the current experimental settings. This analysis was performed for each channel; additionally, we performed a statistical test to evaluate the difference in ERPs between the target and nontarget conditions. Averaged EEG time-series data across trials (within the same condition) for each participant were divided into 20-ms blocks for each channel. In each block, the averaged amplitude value was calculated for each participant. Statistical significance was examined for the amplitude using the Wilcoxon signed-rank test, which is a nonparametric method for repetitive measurements [10,11], and the threshold for statistical significance was set at *p* = 0.05. The ERP analysis was performed using Python and MNE-Python (version 1.2.3 [12]; Python Software Foundation, Wilmington, Delaware, United States). The statistical analysis was performed using SciPy (version 1.9.3).

### 2.7 Classification

We performed a binary classification analysis to evaluate how much EEG data during the oddball paradigm classified the target and nontarget trials; a linear support vector machine (SVM) was used [13]. The classification accuracy was calculated using nested cross-validation [14]. The inner and outer loops of the nested cross-validation were four- and five-fold, respectively. The best cost parameter *C* was searched from 10^−6^ to 10^3^, with an interval of 10^1^. In the 1-Target experiment, the number of target and nontarget trials was not equal (the nontarget was four-fold larger than the target); therefore, we calculated the balanced accuracy [15]. In the 2-Target experiment, the number of target and nontarget trials was equal; in this case, the balanced accuracy was the same as the ordinal accuracy. The accuracy when the input data were averaged across trials (1–5 trials) was also calculated [16]. The trials to be averaged were selected pseudorandomly. Statistical tests were performed using the Friedman test to check the statistical significance, along with the number of trial averages. To verify the performance in a practical setting, we also evaluated the accuracy for selected region of interest (ROI) and single channel (Cz). The ROI was selected from the channels excluding frontal regions (FT7, FC5, FC3, FC1, FCz, FC2, FC4, FC6, FT8, T7, C5, C3, C1, Cz, C2, C4, C6, T8, TP7, CP5, CP3, CP1, CPz, CP2, CP4, CP6, TP8, P9, P7, P5, P3, P1, Pz, P2, P4, P6, P8, P10, PO7, PO3, POz, PO4, PO8, O1, Pz, P2, and Iz) because increased ERPs were observed from these 47 channels (see results). The classification analysis was performed by Python and Scikit-learn (version 1.2.0) [17].

### 2.8 Half session analysis for habituation effects

We also performed session-split analysis to investigate habituation effects, that is, whether ERPs and classification accuracy decreased with sessions. We divided all data into the first half (1–10) and the latter half (11–20) of the sessions. For each half of the sessions, we performed ERP analysis and classification, as described above. Note that the target sound was different in the first and the latter halves of the sessions in the 2-Target experiment. Statistical significance between the first and the latter halves was examined for the amplitude of the Cz channel and the accuracy for single trial (without trial averaging) using the Wilcoxon signed-rank test. The threshold for statistical significance was set at *p* = 0.05.

## 3 Results

### 3.1 ERP analysis

**Fig. 2:**
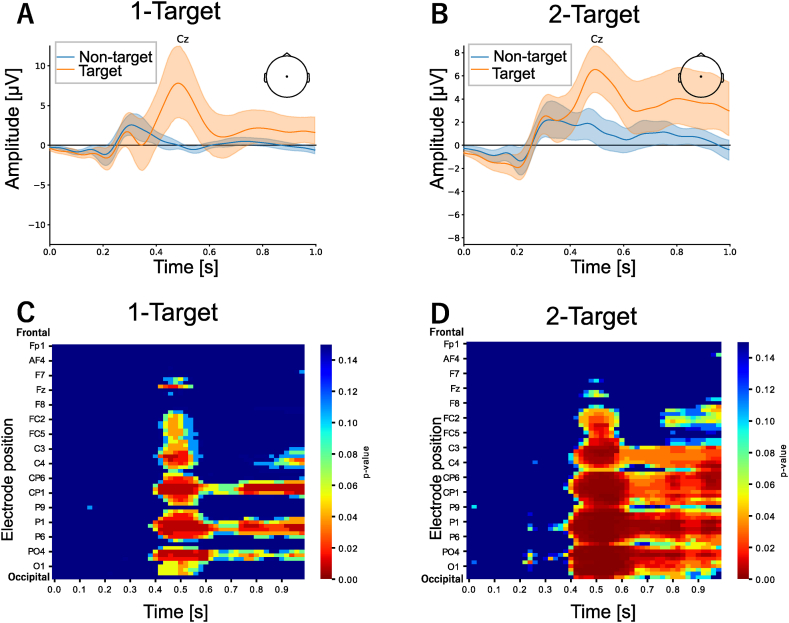
ERP analysis, (A and B) The upper panels show averaged event-related potentials for the Cz channel. Target and nontarget data are shown by orange and blue lines, respectively. The shaded area indicates the confidence interval. (C and D) The lower panels show *p*-values calculated using the Wilcoxon signed-rank test. The color bar indicates the level of *p*-values. Electrode positions are aligned from the frontal (upper) to occipital areas (lower).

To evaluate ERP responses in an oddball paradigm using auditory volume levels, we examined the averaged EEG time-series data for target and nontarget trials at the Cz channel (Fig. 2A for experiment 1, Fig. 2B for experiment 2). We found large positive responses around 400-ms after stimulus onset (P300) for the target trials in both experiments. We also found statistically significant differences between the target and nontarget trials, mainly for the central, parietal, and occipital channels (Fig. 2C and D). These results were observed in both experiments. Based on this result, 47 channels from central, parietal, and occipital channels were selected for the classification with the selected ROI.

### 3.2 Classification accuracy

Table 1, Table 2 show the classification accuracy, along with the average number of trials for the 1-Target and 2-Target experiments. In the 1-Target experiment, the accuracy reached 0.809 without trial averaging, and 0.909 with 5-trial averaging. In the 2-Target experiment, the accuracy was slightly lower than in the 1-Target experiment, reaching 0.695 without trial averaging, and 0.759 with 4-trial averaging. Statistical tests using the Friedman test revealed significant effects of trial averaging (Q = 34.0, *p* < 0.05). Next, we evaluated the accuracy of the selected ROIs in the 2-Target experiment (Table 3). For the selected ROIs, the mean accuracy without trial averaging was 0.677 and a maximum of 0.723 case of 4-trial averaging. Thus, reducing the number of channels from 64 to 47 did not affect the accuracy while we found the significant difference for the result without trial averaging compared with data with all the channels (T = 44, *p* < 0.05). However, when using a single channel (Cz), the accuracy decreased by approximately 10 % compared to that when using all channels (Table 4). The significant difference was observed for the result without trial averaging compared with data with all the channels (T = 44, *p* < 0.05).

**Table 1:** Accuracy of the 1-Target experiment.

Participant no.	The number of trials averaged				
	1	2	3	4	5
1	0.74	0.71	0.79	0.80	0.86
2	0.74	0.69	0.85	0.89	0.93
3	0.84	0.80	0.84	0.92	0.91
4	0.74	0.69	0.79	0.83	0.91
5	0.90	0.88	0.92	0.91	0.93
6	0.86	0.83	0.87	0.96	0.93
7	0.87	0.87	0.90	0.96	0.92
Average	0.809	0.778	0.848	0.892	0.909
SD	0.065	0.079	0.045	0.057	0.024

SD: standard deviation.

**Table 2:** Accuracy of the 2-Target experiment.

Participant no.	The number of trials averaged				
	1	2	3	4	5
1	0.66	0.67	0.62	0.74	0.78
2	0.65	0.68	0.64	0.73	0.59
3	0.65	0.57	0.62	0.63	0.71
4	0.63	0.63	0.64	0.69	0.65
5	0.81	0.80	0.88	0.93	0.92
6	0.73	0.71	0.72	0.78	0.70
7	0.72	0.65	0.78	0.74	0.74
8	0.71	0.73	0.71	0.77	0.83
9	0.67	0.63	0.64	0.73	0.69
10	0.72	0.72	0.77	0.85	0.83
Average	0.695	0.678	0.712	0.759	0.744
SD	0.051	0.061	0.080	0.079	0.093

SD: standard deviation.

**Table 3:** Accuracy of the 2-Target experiment with selected ROI (47 channels).

Participant no.	The number of trials averaged				
	1	2	3	4	5
1	0.63	0.67	0.64	0.72	0.68
2	0.63	0.64	0.62	0.74	0.64
3	0.60	0.47	0.60	0.60	0.66
4	0.61	0.64	0.63	0.62	0.54
5	0.80	0.76	0.86	0.81	0.90
6	0.69	0.71	0.71	0.78	0.75
7	0.69	0.61	0.82	0.71	0.79
8	0.73	0.70	0.72	0.75	0.83
9	0.66	0.59	0.73	0.67	0.64
10	0.73	0.74	0.77	0.83	0.78
Average	0.677	0.653	0.710	0.723	0.721
SD	0.060	0.080	0.084	0.072	0.102

SD: standard deviation.

**Table 4:** Accuracy of the 2-Target experiment with Cz channel.

Participant no.	The number of trials averaged				
	1	2	3	4	5
1	0.66	0.61	0.62	0.76	0.76
2	0.59	0.61	0.64	0.65	0.52
3	0.53	0.44	0.43	0.47	0.58
4	0.54	0.54	0.58	0.53	0.33
5	0.62	0.56	0.62	0.73	0.78
6	0.56	0.57	0.63	0.65	0.47
7	0.58	0.59	0.66	0.61	0.67
8	0.56	0.47	0.51	0.52	0.57
9	0.57	0.49	0.59	0.43	0.55
10	0.73	0.49	0.59	0.43	0.55
Average	0.594	0.561	0.605	0.619	0.602
SD	0.058	0.079	0.085	0.126	0.141

SD: standard deviation.

### 3.3 Half-session analysis

**Fig. 3:**
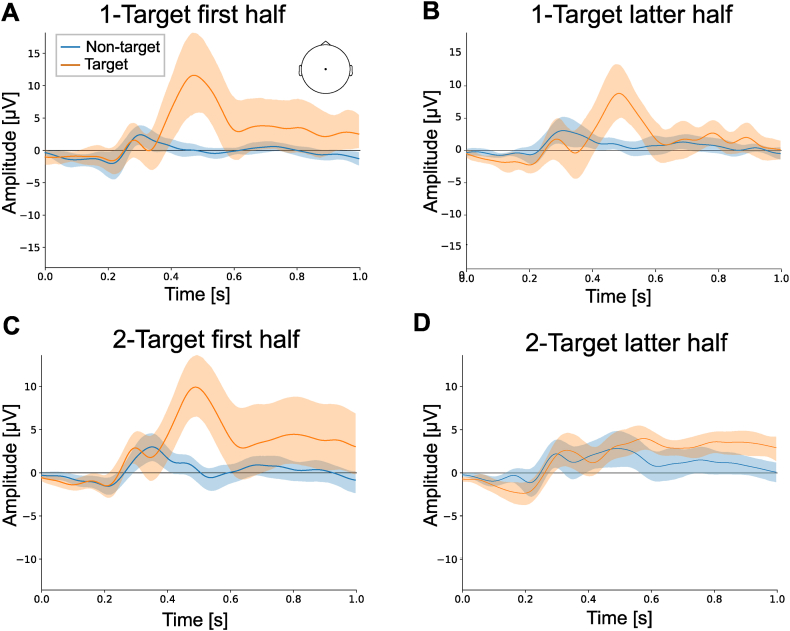
ERP analysis for half-session analysis, (A and B) The upper panels show the averaged event-related potentials for the Cz channel for the first half of the sessions (A) and the latter half of the sessions (B) for the 1-Target experiment. (C and D) The lower panels show averaged event-related potentials for the Cz channel for the first half of the sessions (C) and the latter half of the sessions (D) for the 2-Target experiment. The same colors and legends were used, as shown in Fig. 2. -

To examine the effects of habituation, we performed a half-session analysis in which the data were divided into the first and latter half of the sessions. We reported classification accuracies for single and 4-trial averaging only as a representative result (Table 5, Table 6). In the 1-Target experiment, we found similar results for each half-session. For ERP analysis, the participant-averaged amplitude for the target stimulus was 11.6 μV in the first half (Fig. 3A), while it decreased to 8.8 μV in the latter half (Fig. 3B). No statistically significant difference was observed (T = 27, *p* = 0.16). The classification accuracy for the latter half of the sessions also decreased by 5.7 % (Table 5) but no significant difference was observed (T = 16, *p* < 0.12 for without trial averaging). Similar accuracies were observed when using 4-trial averaging. Thus, the performance was slightly changed in the latter half of the sessions, but not so large. On the other hand, a large difference between the first and the latter half of the sessions was observed in the 2-Target experiment. We found decreasing amplitudes for the target stimulus in the latter half of the sessions (4.0 μV, Fig. 3D) compared to the first half (9.9 μV, Fig. 3C). The statistically significant difference was observed (T = 52, *p* < 0.05). Similarly, the accuracy for the latter half of the session in the 2-Target experiment significantly decreased by 9.9 % compared to that for the first half of the sessions (Table 6, T = 52, *p* < 0.05 for without trial averaging), whereas the accuracy was almost the same as the results when using all the data (Table 2).

**Table 5:** Accuracy of the half-session analysis in the 1-Target experiment.

Participant no.	First half		Latter half	
	The number of trials averaged		The number of trials averaged	
	1	4	1	4
1	0.55	0.48	0.72	0.63
2	0.83	0.92	0.77	0.81
3	0.69	0.83	0.64	0.58
4	0.70	0.56	0.70	0.78
5	0.84	1.00	0.73	0.92
6	0.79	0.92	0.63	0.92
7	0.83	0.58	0.64	0.75
Average	0.747	0.756	0.690	0.770
SD	0.099	0.194	0.050	0.121

SD: standard deviation.

**Table 6:** Accuracy of the half-session analysis in the 2-Target experiment.

Participant no.	First half		Latter half	
	The number of trials averaged		The number of trials averaged	
	1	4	1	4
1	0.73	0.89	0.64	0.70
2	0.78	0.80	0.63	0.52
3	0.65	0.73	0.73	0.72
4	0.74	0.83	0.60	0.62
5	0.87	0.92	0.82	0.80
6	0.83	0.94	0.66	0.70
7	0.75	0.83	0.70	0.70
8	0.87	0.86	0.72	0.80
9	0.78	0.91	0.67	0.66
10	0.90	0.97	0.74	0.84
Average	0.790	0.868	0.691	0.706
SD	0.073	0.069	0.061	0.089

SD: standard deviation.

## 4 Discussion

Automatic volume control using the BCI is required to detect the sound pressure level that the user intends to change. As a first step, this study examined an oddball paradigm using changes in the sound pressure level of white noise stimuli toward BCIs for volume control. ERP analysis only showed increased positive responses for the target trials, which were likely to be P300. This suggests that changes in the sound pressure level can be detected as neural responses reflected by EEG. The general setting of volume control requires two types of commands: up and down. Thus, this is a binary or a three-class classification problem. Therefore, it may be possible to use existing BCIs for volume control because such classification problems are common in BCIs. However, if we use stimuli or tasks irrelevant to the auditory signal (e.g., left-hand motor imagery to volume up and right-hand imagery to volume down), it is non-intuitive and difficult to use in practical application. On the other hand, our approach directly uses neural responses to auditory signals, and volume control is easily achieved by paying attention to sound. To the best of our knowledge, this is the first study to demonstrate the possibility of automatic volume control using brain signals in an intuitive manner. Currently, our study did not establish automatic volume control yet. However, we anticipate that fundamental results from this study can be extended to practical BCI application with natural human intention.

For classification, we confirmed a high accuracy for the 1-Target paradigm, suggesting that volume control using a BCI might be possible for simple (1-command) settings. However, volume control usually requires two commands (up and down); thus, the 2-Target paradigm would be preferable. Our results for the 2-Target experiment demonstrated the highest average accuracy of 0.759 (4-trial averaging), which is relatively low compared to that for the 1-Target experiment (0.909 at the highest average accuracy). This could be due to the emergence of ERP or P300. P300 is likely the main contributor to the classification, and is involved in cognitive aspects such as comparison with previously presented stimuli [18]. In this case, the previously presented stimuli to be compared were probably the frequent stimuli, but not infrequent nontarget stimuli. Therefore, it might be more difficult to detect the target stimulus from the nontarget in the 2-Target experiment, than from two types of sounds in the 1-Target experiment. This may affect the accuracy of the classification [18]. Another possibility is that it is more difficult to detect small sound-level target stimuli than large ones. We have no quantitative behavioral data for the difficulty of the task. However, many participants reported that a small volume (50 dB) was more difficult than a large one (70 dB). When we performed the half-session analysis, the ERP amplitudes decreased (Fig. 3D), and degraded accuracy (Table 6) were observed in the latter half of the sessions, where the target stimulus was a small sound level (50 dB). As shown in the results of the 1-Target experiment, there might be habituation effects; ERP amplitudes were slightly decreased in the latter half of the sessions (Fig. 3A and B). However, according to the larger difference in ERP amplitudes and classification accuracy between the first and latter half of the sessions in the 2-Target experiment, the target amplitude difference may be more important for classification. It is known that the amplitude of auditory evoked responses is correlated with the sound pressure level, called loudness-dependent auditory evoked potentials [[19], [20], [21]], including P300 [22,23]. Because of this characteristic, it may be easier to detect large volumes and construct an automatic control system for volume reduction.

The single trial accuracy (0.695) in our study was larger than in the previous study (0.602) [8], and the corresponding information transfer rates [24] were 1.46 bits/min in our study and 0.48 bits/min in the previous study [8]. One of the reasons for this improvement is that the ratio of the frequent stimulus (60 dB in our study) was lower (60 %) than in the previous study (71.4 %). However, this improvement in accuracy is a promising result, even when reducing the frequent stimulus and increasing the target stimulus. Thus, although there are several differences between the two studies (e.g., experimental paradigm, difficulty, and classification algorithm), our results suggest the possibility of detecting changes in sound pressure levels. Future studies may improve the accuracy by evaluating the paradigm and analysis, which we did not examine.

To construct a real application without any unintended changes in volume for both up and down, it is necessary to improve the accuracy of automatic control in a single trial. In our study, we used SVM, a classical machine learning algorithm for classification. Many previous studies have proposed methods to improve classification accuracy. For example, independent component analysis [25] or convolutional neural network as a classifier [26,27] can be used to improve classification accuracy. Although investigating the best method is beyond the scope of the current study, these methods can be used to improve the single-trial classification accuracy for practical applications. Furthermore, comparisons between different channel selections (as shown in Table 3, Table 4) suggest that reducing the channels may lead to degradation in performance although this reduction is suitable for real application. This issue should be explored in conjunction with the classifier.

In conclusion, our results suggest a possibility of the auditory BCI for automatic volume control. Since the current study was conducted using white noise stimuli, testing different types of auditory stimuli in more practical situations, including in the clinical setting, should be considered in the future.

## Data availability statement

The data and code associated with this study has been deposited at https://github.com/nambulab-nagaokaut/spl_control_eeg_public.

## Additional information

No additional information is available for this paper.

## CRediT authorship contribution statement

**Riki Kimura:** Conceptualization, Data curation, Formal analysis, Investigation, Methodology, Software, Writing – original draft. **Isao Nambu:** Conceptualization, Funding acquisition, Supervision, Writing – review & editing. **Rui Fujitsuka:** Formal analysis, Investigation, Methodology. **Yoshiko Maruyama:** Methodology, Writing – review & editing. **Shohei Yano:** Conceptualization, Funding acquisition, Methodology, Writing – review & editing. **Yasuhiro Wada:** Conceptualization, Funding acquisition, Writing – review & editing.

## Declaration of competing interest

The authors declare that they have no known competing financial interests or personal relationships that could have appeared to influence the work reported in this paper.
